# Evidence for gene flow from the Gulf of Mexico to the Atlantic Ocean in bonnethead sharks (*Sphyrna tiburo*)

**DOI:** 10.1002/ece3.70334

**Published:** 2024-09-22

**Authors:** Kristina L. Black, Kathy Liu, Jasmin R. Graham, Tonya R. Wiley, Jayne M. Gardiner, Catherine Macdonald, Mikhail V. Matz

**Affiliations:** ^1^ Department of Integrative Biology University of Texas at Austin Austin Texas USA; ^2^ Rosenstiel School of Marine, Atmospheric, and Earth Science University of Miami Miami Florida USA; ^3^ Minorities in Shark Sciences Bradenton Florida USA; ^4^ Havenworth Coastal Conservation Palmetto Florida USA; ^5^ New College of Florida Sarasota Florida USA; ^6^ Field School Coconut Grove Florida USA

**Keywords:** bonnethead shark, connectivity, demographic modeling, gene flow, migration, population structure

## Abstract

Gene flow is important for maintaining the genetic diversity required for adaptation to environmental disturbances, though gene flow may be limited by site fidelity in small coastal sharks. Bonnethead sharks (*Sphyrna tiburo*)—a small coastal hammerhead species—demonstrate site fidelity, as females are philopatric while males migrate to mediate gene flow. Consequently, bonnetheads demonstrate population divergence with distance, and Atlantic populations are genetically distinct from those of the Gulf of Mexico. Indeed, Florida forms a vicariant zone between these two bodies of water for many marine species, including some sharks. However, while bonnetheads are expected to have limited dispersal, the extent and rate of bonnethead migration remain uncertain. Thus, we aimed to determine their dispersal capacity by evaluating connectivity between disparate populations from the Gulf of Mexico and Atlantic Ocean. Using 10,733 SNPs derived from 2bRAD sequences, we evaluated genetic connectivity between Tampa Bay on the Gulf Coast of Florida and Biscayne Bay on the Atlantic coast of Florida. While standard analyses of genetic structure revealed slight but significant differentiation between Tampa Bay and Biscayne Bay populations, demographic history inference based on the site frequency spectrum favored a model without divergence. However, we also estimate that if population divergence occurred, it would have been recent (between 1500 and 4500 years ago), with continuous unidirectional gene flow from Tampa Bay to Biscayne Bay. Our findings support the hypothesis that bonnetheads can migrate over relatively large distances (>300 miles) to find mates. Together, these results provide optimism that under proper management, a small‐bodied globally endangered shark can undergo long migrations to sustain genetic diversity.

## Graphical abstract

Genomic data from bonnethead sharks reveals connectivity between the Gulf and Atlantic coasts of Florida, a zone that forms a genetic barrier for some larger shark species. Our demographic model also revealed rapid population growth and gene flow from the Gulf Coast to the Atlantic Coast, suggesting that sharks may migrate south for mating. These findings provide novel insight into the migratory behavior of small‐bodied sharks and optimistic implications for the conservation of this globally endangered species under proper management.

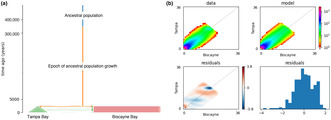

## 1 INTRODUCTION

Sharks are highly exploited around the world, and recovery is limited by low reproductive rates (Dulvy et al., 2017) and fishing pressure (Ferretti et al., 2010; Worm et al., 2024). However, many sharks migrate long distances (Speed et al., 2010) which enhances gene flow that is vital for maintaining genetic diversity and aiding adaptation to environmental change. For shark populations to persist, there is a growing need to understand population connectivity and evolutionary processes that affect them (Domingues et al., 2018). Bonnethead sharks (*Sphyrna tiburo*) are a small‐bodied coastal hammerhead species that is a common seasonal resident around the southern coasts of North America, including the western Atlantic Ocean and Gulf of Mexico. Despite being abundant in northwest and west central Atlantic waters due to management, populations in the Caribbean Sea, southwest Atlantic Ocean, and east central and southwest Pacific Ocean are largely depleted and severely fragmented, leading to the species being assessed as globally Endangered (IUCN, 2023).

Bonnetheads have a small home range (Driggers et al., 2014; Heupel et al., 2006) and demonstrate cryptic genetic divergence in the Atlantic, Caribbean, and the eastern Pacific (Aroca et al., 2022; Gonzalez et al., 2019). There appear to be geographic barriers to gene flow between Atlantic and Caribbean populations leading to speculation about cryptic speciation (Fields et al., 2016). Although these distinct genetic lineages occasionally hybridize, they should be managed as separate stocks with unique management needs, especially since the eastern Pacific lineage may have been extirpated from the Gulf of California in recent years (Pérez‐Jiménez, 2014). Bonnethead populations also exhibit genetic divergence across the Atlantic and Gulf coasts of Florida (Díaz‐Jaimes et al., 2021; Escatel‐Luna et al., 2015; Fields et al., 2016; Portnoy et al., 2015). However, while migration in other hammerhead species is well‐studied (Gallagher & Klimley, 2018), little is known about migration in bonnethead sharks. To fill in these knowledge gaps, molecular approaches can be employed to investigate genetic connectivity and dispersal.

For many marine species, the Florida peninsula presents a barrier to gene flow between Gulf and Atlantic coast populations (Neigel, 2009). This has been observed in coastal shark species; studies using mtDNA and microsatellites in finetooth sharks (*Carcharhinus isodon*; Portnoy et al., 2016) and genomic studies of blacknose sharks (*C. acronotus*; Dimens et al., 2019) and blacktip sharks (*C. limbatus*; Swift et al., 2023) detected population divergence between Gulf and Atlantic coast sites (Dimens et al., 2019). However, recapture studies suggest that South Florida's geography does not prevent blacknose shark movement, with some migrants captured moving from the Gulf to the Atlantic (Dimens et al., 2019). Consequently, the Florida Keys are considered a putative seasonal mixing zone. Conversely, studies of larger‐bodied coastal sharks, such as sandbar (*C. plumbeus*; Portnoy et al., 2010) and lemon sharks (*Negaprion brevirostris*; Ashe et al., 2015), show genetic connectivity between Gulf and Atlantic populations. Gullivan Bay on Florida's southwestern tip exhibited higher haplotype diversity in lemon sharks than higher latitudes, supporting southern Florida as a mixing zone (Ashe et al., 2015). Notably, these larger sharks are more capable of long‐distance migrations. Sandbar sharks mature at 123–190 cm (Baremore & Hale, 2012; Ebert et al., 2013), and lemon sharks at 224–239 cm (Brown & Gruber, 1988; Ebert et al., 2013). Whereas the genetically divergent species are smaller, finetooth sharks mature at 96–100 cm (Higgs et al., 2020), blacknose sharks at 80–113 cm (Carlson et al., 1999), and blacktip sharks at 105–207 cm (Baremore & Passerotti, 2013; Rigby et al., 2021). Bonnethead sharks are even smaller—maturing at 56–77 cm in the Gulf of Mexico (Frazier et al., 2023)—likely resulting in more limited dispersal for bonnethead sharks (Speed et al., 2010).

Genetic barriers between populations may lead to local adaptation or evolution of new ecotypes or morphotypes. Indeed, morphological differences were detected between bonnethead populations from Tampa Bay and Florida Bay, both on the Gulf side of the Florida Peninsula but over 300 km apart (Lombardi‐Carlson et al., 2003). Bonnetheads from these disparate sites varied in developmental measures, such as age of maturity (Parsons, 1993) and reproductive rates (Cortés & Parsons, 1996). Other studies found that size at maturity differs between bonnethead populations from the Gulf of Mexico and the Atlantic (Frazier et al., 2014) and that size at maturity also increases with latitude (Lombardi‐Carlson et al., 2003). Moreover, female bonnethead sharks from Tampa Bay were significantly larger than female bonnethead sharks from the Florida Keys (Parsons, 1993). And in populations from the western Atlantic and eastern Pacific, morphological differences were associated with divergence in mtDNA (Aroca et al., 2022), though these populations may even represent cryptic species.

Although migration behavior of bonnetheads remains enigmatic, recapture studies have confirmed seasonal abundance of populations in multiple bays. April to November recaptures in three bays of Florida suggested that individuals are seasonal residents in the same bays (Pine Island Sound—Heupel et al., 2006; Apalachicola Bay and St. George Sound—Peterson, 2021), and recaptures in South Carolina estuaries over multiple years confirmed site fidelity (Driggers et al., 2014). However, lower catch rates on their respective bays during the winter months imply that populations migrate elsewhere (Peterson, 2021), though where they go remains unknown. One study of bonnethead populations from South Carolina recaptured individuals in the winter as far south as Florida (Driggers et al., 2014), suggesting the possibility of a southward migration that aligns with that seen in other shark species (Ashe et al., 2015; Dimens et al., 2019; Kajiura & Tellman, 2016). However, a large bonnethead recapture study that sampled both the Gulf and Atlantic coasts of Florida did not observe any individuals who traveled between coasts (Bethea & Grate, 2013). Thus, while at least more northern populations of bonnetheads likely migrate south during the winter, the exchange between Gulf and Atlantic populations around the southern tip of Florida has not yet been confirmed. Indeed, if sharks migrate to southern Florida to reproduce, this might maintain gene flow between Gulf and Atlantic populations.

Previous genetic studies found that bonnethead populations across the Gulf Coast of Florida demonstrate genetic connectivity as far south as the Florida Keys, as demonstrated in mtDNA (Fields et al., 2016; Portnoy et al., 2015), microsatellite (Díaz‐Jaimes et al., 2021), and genomic data (Portnoy et al., 2015). However, divergent mtDNA and genomes were identified on the Gulf and Atlantic coasts of Florida (Díaz‐Jaimes et al., 2021; Escatel‐Luna et al., 2015; Fields et al., 2016; Portnoy et al., 2015). However, while genomic data from Gulf Coast sites are well represented from northern Florida down to the Florida Keys (Díaz‐Jaimes et al., 2021; Portnoy et al., 2015), the Atlantic sites in all these studies were sampled from central Florida or further north, from which migration to the Gulf Coast is unlikely for a small‐bodied shark species known to have a small seasonal home range. Moreover, the southern Atlantic coast of Florida is underrepresented in genetic studies of bonnetheads but might better capture possible connectivity from the Gulf Coast. Thus, genomic studies from southern Florida are needed to capture gene flow that connects populations from both coasts.

Using genomic data, we aimed to evaluate genetic connectivity between bonnethead shark populations in Tampa Bay on the Gulf coast of Florida and Biscayne Bay on the Atlantic coast of Florida. Biscayne Bay has not been represented in prior genetic studies of bonnetheads, despite being located at the southeastern tip of the Florida peninsula. If southern Florida is indeed a mixing zone for migratory sharks, we hypothesized that there may be gene flow between the two sites. We explored the genetic structure of populations from both bays and modeled demographic changes through time. Due to morphological divergence detected in previous studies, we also investigated genetic associations with morphological differences among our samples. Lastly, we discuss how our findings contribute to our growing understanding of migratory behavior and genetic barriers for small coastal sharks.

## 2 METHODS

### 2.1 Sample collection and DNA sequencing

From May to November 2022, 108 fin clips were collected from bonnethead sharks (*Sphyrna tiburo*) in the greater Tampa Bay (including Terra Ceia Bay and Manatee River) on the Gulf coast (27° N 82° W) and Biscayne Bay on the Atlantic coast (25° N 80° W) of Florida (*n* = 24 and 94, respectively). For each sampled individual, sex and morphometrics including precaudal length, fork length, and stretched total length were recorded, and individuals were assigned a life stage (immature, mature) based on clasper calcification (males) or 50% size‐at‐maturity (females: Lombardi‐Carlson et al., 2003). Sampling activities in Tampa Bay were conducted in accordance with Florida Wildlife Conservation Commission Special Activities License SAL‐1666‐SRP. All protocols for the handling and use of animals were approved by the Institutional Animal Care and Use Committee at the University of South Florida (protocol # W IS00004541). Data collection in Biscayne Bay was permitted by the Florida Fish and Wildlife Conservation Commission (SAL‐23‐1798‐SRP) and approved by the University of Miami's Institutional Animal Care and Use Committee (protocol #20‐044).

Samples were preserved in DMSO or 95% ethanol and stored at −25°C. Genomic DNA was isolated using a modified phenol‐chloroform protocol (Data S1), and 2bRAD libraries were prepared to sequence sparse restriction sites across the entire genome, which is ideal for profiling genetic variation in natural populations (Wang et al., 2012, the most current lab protocol and data processing pipeline are hosted at: https://github.com/z0on/2bRAD_denovo). In brief, samples, including 10 randomly chosen technical replicates, were normalized to a concentration of 25 ng/μl before digestion with the BcgI restriction enzyme. Adapters were then ligated to digested DNA, followed by amplification with iTru primers. The PCR product was purified with magnetic beads and pooled. Then the multiplexed libraries were size selected at 170–190 bp and subsequently sequenced across three lanes of the Illumina NovaSeq SR100 platform at the Genomic and Sequencing Analysis Facility at the University of Texas at Austin. Reads were demultiplexed and deduplicated using 2bRAD‐specific script (https://github.com/z0on/2bRAD_denovo/trim2bRAD_2barcodes_dedup.pl), trimmed with Cutadapt (Martin, 2011) to remove low‐quality read ends, and then mapped to the great hammerhead genome (*Sphyrna mokarran*, NCBI accession #: GCA_024679065.1) using bowtie2 (Langmead & Salzberg, 2012). To filter samples by sequencing depth, individuals with less than a quarter of sites at 5× or greater coverage were discarded. After confirming alignment of technical replicates, only replicates with highest sequencing depth were retained. To filter sites, we used the software ANGSD (i.e., Analyzing Next Generation Sequencing Data; Korneliussen et al., 2014) to remove low‐quality SNPs (with >0.1% chance of sequencing error and >0.1% chance of erroneous mapping), sites not genotyped in at least 75% of individuals (to remove contaminants), and sites with a minor allele frequency < 0.025. After filtering, we derived identity‐by‐state (IBS) genetic distances between all samples from both bays. We also derived genotype likelihoods, which contain probabilities of unobserved genotypes in sparse genomic data that are useful for downstream inference of relatedness, ancestry, and inbreeding. We evaluated relatedness among samples (via maximum likelihood estimator NgsRelate; Korneliussen & Moltke, 2015) using the pairwise relatedness estimate (Hedrick & Lacy, 2015), which is the main summary statistic based on the nine Jaquard coefficients (Jacquard, 1974) that NgsRelate computes. Estimates of the probability of shared alleles between individuals were summarized into a pairwise relatedness distance matrix of all samples from both bays.

### 2.2 Population structure

We explored population structure in a principal coordinates analysis (PCoA) of the genetic distance matrices (IBS and relatedness). Significance of population differentiation in the PCoA space was assessed using permutational ANOVA (PERMANOVA implemented in function *vegan::adonis2* in R; Oksanen et al., 2020). This function is robust to sampling error from unbalanced sample sizes as random independent permutations shuffle samples 999 times between bins of the same size. To support conclusions about population structure inferred from PCoA, we also evaluated the possibilities of two or more genetically distinct ancestries within the sample set by examining admixture scenarios *k* = 2–4. For each scenario of *k* (i.e., putatively different ancestral populations), admixture proportions were estimated from genotype likelihoods using maximum likelihood estimator (NGSadmix; Skotte et al., 2013). However, without a statistical criterion to determine the value of *K*, variation in admixture proportions across individuals was simply used to visualize the effect of multiple ancestral populations rather than to estimate the number of ancestral populations. Additionally, individual heterozygosity was derived using ANGSD (on all sites with at least 10× coverage and mapping quality with <0.01% errors) by calculating the number of heterozygous genotypes in single‐sample site frequency, then dividing by the total number of sites for each sample to estimate the proportion of heterozygous sites. Per‐individual inbreeding coefficients were estimated from genotype likelihoods using a maximum likelihood estimator (ngsF; Vieira et al., 2013). Lastly, global and per‐site *F*_ST_ was estimated using priors derived from the two‐dimensional site frequency spectrum (see next section).

### 2.3 Demographic history

To investigate population structure in more detail and evaluate directionality of migration between the bays, we inferred the joint demographic history of Tampa Bay and Biscayne Bay populations using the 2D site frequency spectrum (SFS). The SFS is essentially a histogram of allele frequencies of single‐nucleotide variants in two populations, from which the demographic history of the populations can be inferred (Gutenkunst et al., 2009). We inferred the SFS using ANGSD, considering all sites with a mapping and base call quality score > 20 (i.e., probability of error < 0.01), genotyped in at least 75% of samples from both Tampa and Biscayne Bays, with an additional MaxHetFreq filter to remove sites with excessive heterozygosity likely representing paralogous loci.

We implemented GADMA (“Genetic Algorithm for Demographic Model Analysis,” Noskova et al., 2020) with 96 optimizations to infer the demographic model that best fits the distribution of allele frequencies at both bays. GADMA simulates the SFS through global optimization using a composite likelihood scheme, which determines the likelihood of obtaining the observed spectrum given an expected spectrum. The likelihood functions simulate genetic drift with mutation and crossover to infer the structure and parameters of the demographic model. Our implementation of GADMA used the *moments* engine (Jouganous et al., 2017) to fit the model, which relies on ordinary differential equations to find the likelihood peak. This approach, however, assumes that the populations being compared are distinct and does not consider a model with no population split. Thus, this method was only implemented after populations demonstrated significant differences in PCoA. Starting with the simplest model structure (i.e., the least number of population splitting events and time intervals for population size change), the parameters of splitting events and time intervals were adjusted to improve the composite likelihood. After optimizing parameters, complexity of model structure increased until the optimization was reached, and the model with the greatest likelihood best fit the observed SFS. To avoid overfitting, composite likelihood Akaike information criterion (CLAIC) was compared among final models with different numbers of parameters. The final structure of the best‐fit demographic model indicated the number of time intervals before and after each splitting event. The final parameters indicated the length of time, the effective population size at the end of the time interval, the dynamics of population size change, and migration between any populations during that interval.

To account for the discrepancy in sample sizes between the two populations, we adjusted the Biscayne Bay sample by projecting its site frequency spectrum (SFS) to match the size of the Tampa Bay sample set. Consequently, both populations were projected down to 36 genomes each. To maintain a conservative approach in the demographic analysis and reduce the number of parameters to be inferred, we did not differentiate alleles as ancestral or derived, opting to use a “folded” SFS. We used the mutation rate (μ) as in the great hammerhead, 3.92 × 10^−9^ per base per generation (Martin, 1999; Stanhope et al., 2023) as it is the most genetically similar species available in the literature. We also assumed a generation time (*G*) of 4 years, based on the time to sexual maturity in bonnetheads from Tampa Bay (Cortés & Parsons, 1996; Frazier et al., 2023). Using the *moments* engine (Jouganous et al., 2017), we specified the initial model with the simplest structure, which included single time intervals before and after any possible divergence. New generations of demographic models were then produced by implementing mutations and crossover between populations until the log‐likelihood could no longer be improved over 100 iterations.

To test the robustness of the optimal model identified by GADMA and enhance the confidence in the optimized model, we conducted an additional model selection analysis to challenge the model structure. Specifically, we tested whether the optimized GADMA model holds up when comparing models with no population split, no migration, or symmetric migration (following methods from (Rippe et al., 2021); https://github.com/z0on/AFS‐analysis‐with‐moments/tree/master). We generated 100 bootstrapped SFS using ANGSD, based on subsampling contigs with replacement, which accounts for possible inflation of confidence due to physical linkage between SNPs within the same contig. We then compared seven simple demographic models that varied based on whether populations split, the number of population growth epochs, and whether the split populations experienced symmetric or asymmetric migration (Table 1). Model selection was performed by running the seven models on 10 bootstrapped SFS with six random restarts. From these 420 runs, the model with the lowest median Akaike Information Criterion (AIC) score among the bootstrap replicates was identified as the best‐fit model. This winning model was subsequently run on all 100 bootstrapped SFS to evaluate uncertainties in derived parameters such as current population size, time since split, and migration rates. By default, the *moments* engine uses relative statistics, calculating population size as the ratio of contemporary to ancient population size (nu) and time as genetic units (T) (Jouganous et al., 2017). However, we utilized theta (θ), the population‐size scaled mutation rate, to derive meaningful parameters. The following conversions were implemented: ancestral effective population size *N*_e_ = θ/4 μ, current effective population size = nu × *N*_e_, migration rate = *M*/2 *N*_e_, and years since population split = 2 T *N*_e_ × *G* (Matz et al., 2018).

**TABLE 1:** Top five best‐fit demographic models by log‐likelihood.

Log‐likelihood	Ancestral Ne	Years since population split	Estimated current Ne of Tampa Bay	Estimated current Ne of Biscayne Bay	Migration rate: BB to TB	Migration rate: TB to BB
−920.78	10,263	1532	158,639	1,026,319	0	4.87e‐04
−920.81	10,354	1558	151,515	1,035,406	0	4.83e‐04
−920.86	13,193	1571	625,934	1,319,378	0	3.79e‐04
−920.86	13,039	1512	544,209	1,303,957	0	3.83e‐04
−920.93	11,421	1527	159,707	1,142,184	0	4.38e‐04

*Note*: The best‐fit model with highest log‐likelihood (top) corresponds to Figure 2. All top models indicate a population split approximately 1500 years ago, population growth in both bays, and asymmetric migration from Biscayne Bay to Tampa Bay.

### 2.4 Genotype associations

We investigated genotype associations with maturity, sex, and morphology in a distance‐based redundancy analysis (dbRDA) using R package *vegan* (Oksanen et al., 2020). dbRDA models linear relationships between meta variables and the IBS genetic distance matrix via permutation tests for the significance of the constrained ordination. Subsequently, forward selection identifies which predictors best summarize the genetic variation. We first evaluated genetic associations with maturity among the whole sample set representing both bays. Then we filtered the data to retain only mature sharks and evaluated genotype‐associations with sex. Lastly, because bonnethead sharks are sexually dimorphic (Kajiura et al., 2005; Lombardi‐Carlson et al., 2003), we looked for genetic associations with morphology (i.e., precaudal length, fork length, and stretched total length) in a partial RDA conditional on sex. Genetic differentiation associated with maturity might imply that a selection event disproportionately impacted one of the age cohorts. Genetic associations with sex might imply that males and females undergo selection in different ways, possibly implying different migration routes for mating. Lastly, genetic associations with morphologic measurements might imply that bonnetheads are evolving into different morphotypes.

## 3 RESULTS

### 3.1 Sequencing and genotyping

On average, we obtained 4,353,449 reads per sample (standard deviation of log10‐transformed read counts = 0.334), of which an average of 3,205,614 reads per sample (log10 s.d. = 0.45) aligned to the great hammerhead genome (Table S1). We first confirmed the integrity of our barcoding scheme by clustering the 10 random technical replicates with their respective samples. After confirming that technical replicates aligned with the correct samples, we removed the replicate with the lowest sequencing depth. The remaining samples were filtered by sequencing depth to retain 89 out of 97 samples (*n* = 20 from Tampa Bay, *n* = 69 from Biscayne Bay). After filtering sites, 10,358 SNPs were retained for producing the final IBS and relatedness distance matrices, and 4,971,503 sites were used to produce the SFS.

### 3.2 Population structure

**FIGURE 1:**
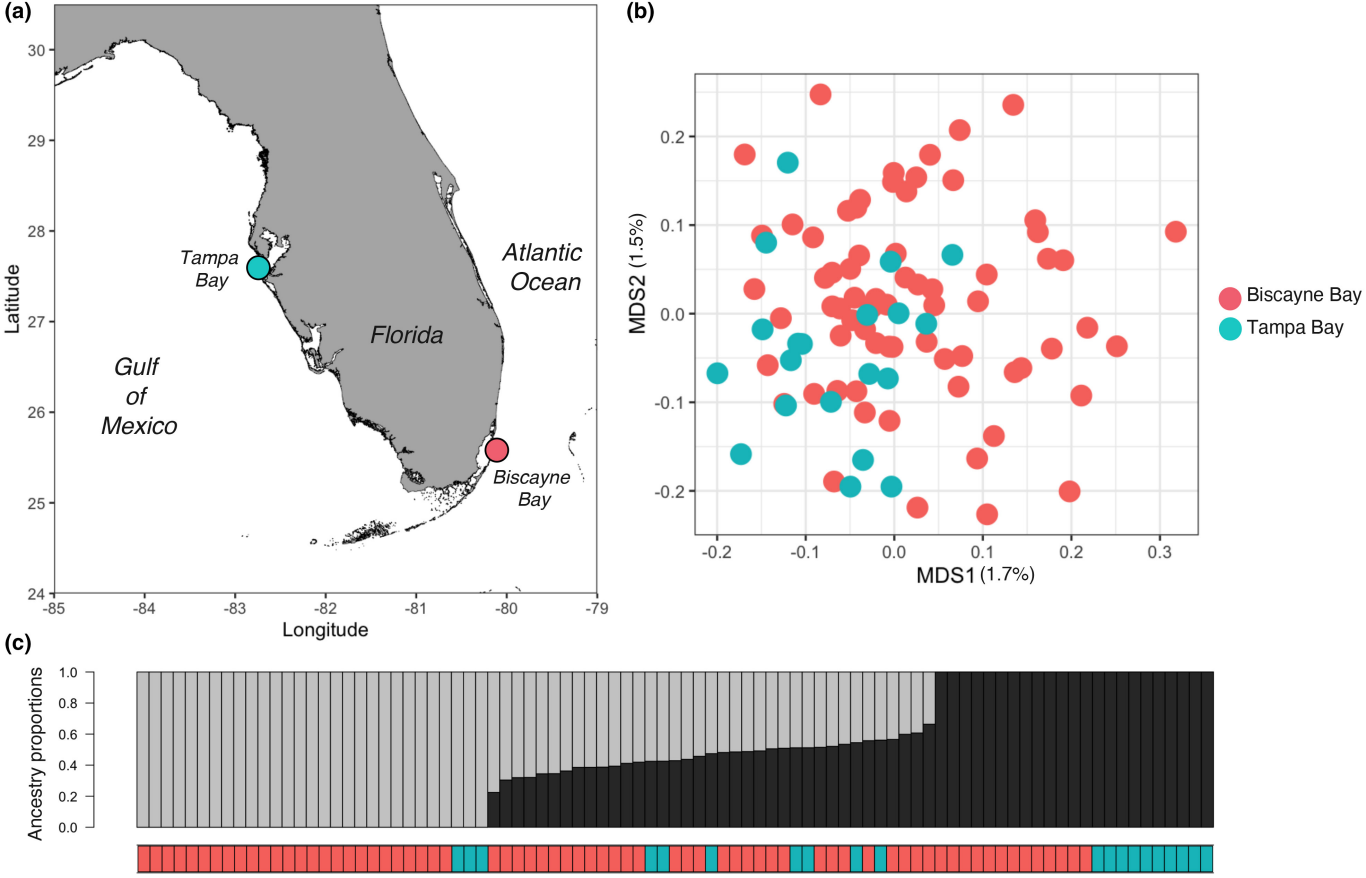
Population structure of bonnethead sharks from Tampa Bay and Biscayne Bay. (a) Locations from which bonnethead sharks were sampled. (b) Principal coordinates analysis showing genetic distance between samples. (c) Admixture bar plot (assuming *k* = 2) shows that Biscayne Bay and Tampa Bay individuals are represented in both ancestry groups, and these two ancestry groups maintain high admixture (i.e., gene flow).

Samples from Tampa Bay and Biscayne Bay demonstrated small but significant genetic differentiation in a PCoA of the IBS matrix (PERMANOVA *R* ^2^ = .01226; *p* = .004, Figure 1b); however, we recognize that increasing the sample size from Tampa Bay could provide a clearer understanding of this population structure. In the simplest admixture scenario (*k* = 2), individuals from both bays comprised both ancestry groups, with many individuals appearing to be admixed (Figure 1c). Evaluating higher *k* values (scenarios considering more ancestral groups) did not reveal a clearer population structure (Figure S2). Overall relatedness among the samples was low, and we did not detect any sibling or parental groups (Figure S1). Despite this, both bays showed very little genetic differentiation (weighted global *F*_ST_ = 0.007394), although the most differentiated genomic sites had a weighted *F*_ST_ = 0.557. Inbreeding was low among all samples from both bays (inbreeding coefficient avg. = 0.002, s.d. = 0.003).

### 3.3 Demographic history

**FIGURE 2:**
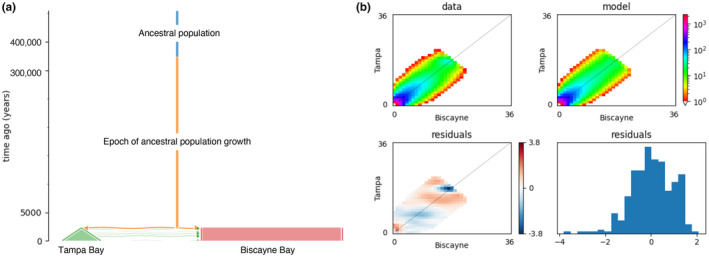
Best‐fit demographic model of bonnethead shark populations from Tampa Bay and Biscayne Bay. (a) The best‐fit demographic model shows that populations split approximately 1500 years ago and maintained continuous unidirectional gene flow from Tampa Bay to Biscayne Bay. Width on the x‐axis shows population size, and the Tampa Bay population demonstrates linear population growth since the split. (b, upper left) The 2D site frequency spectrum (SFS), where color indicates the density of SNPs with similar allele frequencies between Tampa Bay and Biscayne Bay. High‐density SNPs are closest to the diagonal, indicating high migration between the two sites. The best‐fit model (b‐upper right), and the distribution of residuals (b‐bottom) show little difference between observed and predicted SFS.

The top five demographic models determined by GADMA (Table 1) estimated that the ancestral population experienced an epoch of sudden growth approximately 380–400 kya from around 10,000 to approximately 51,000 individuals before the population split (Table S2). Our best‐fit demographic model suggested that, although most single nucleotide variants shared between Tampa Bay and Biscayne Bay populations have similar allele frequencies (Figure 2b), these populations likely diverged as recently as 1500 years ago (Table 1), with continuous unidirectional gene flow from Tampa Bay to Biscayne Bay (Figure 2a and Table 1). The small range of residuals between the observed and predicted SFS (Figure 2b) indicates a good fit of this model to the data. We note that this time of divergence assumes a generation length of 4 years (based on time to maturity, Cortés & Parsons, 1996; Frazier et al., 2023). However, IUCN reports a generation length of 12 years for bonnethead sharks, based on the mean age at which a cohort produces offspring (IUCN, 2023). If we were to assume a generation length of 12 years, then our model would determine that divergence occurred approximately 4500 years ago. After divergence, the Tampa Bay population demonstrated sudden population growth to an effective population size of over 158,000 individuals currently, while the Biscayne Bay population grew to over 1 million individuals. The model indicated that the gene flow is unidirectional, from Tampa Bay to Biscayne Bay, with the estimated proportion of migrants per generation *m* = 4.87 × 10^−4^. Based on the estimated population size of Tampa Bay, this corresponds to 77 migrants per generation.

**FIGURE 3:**
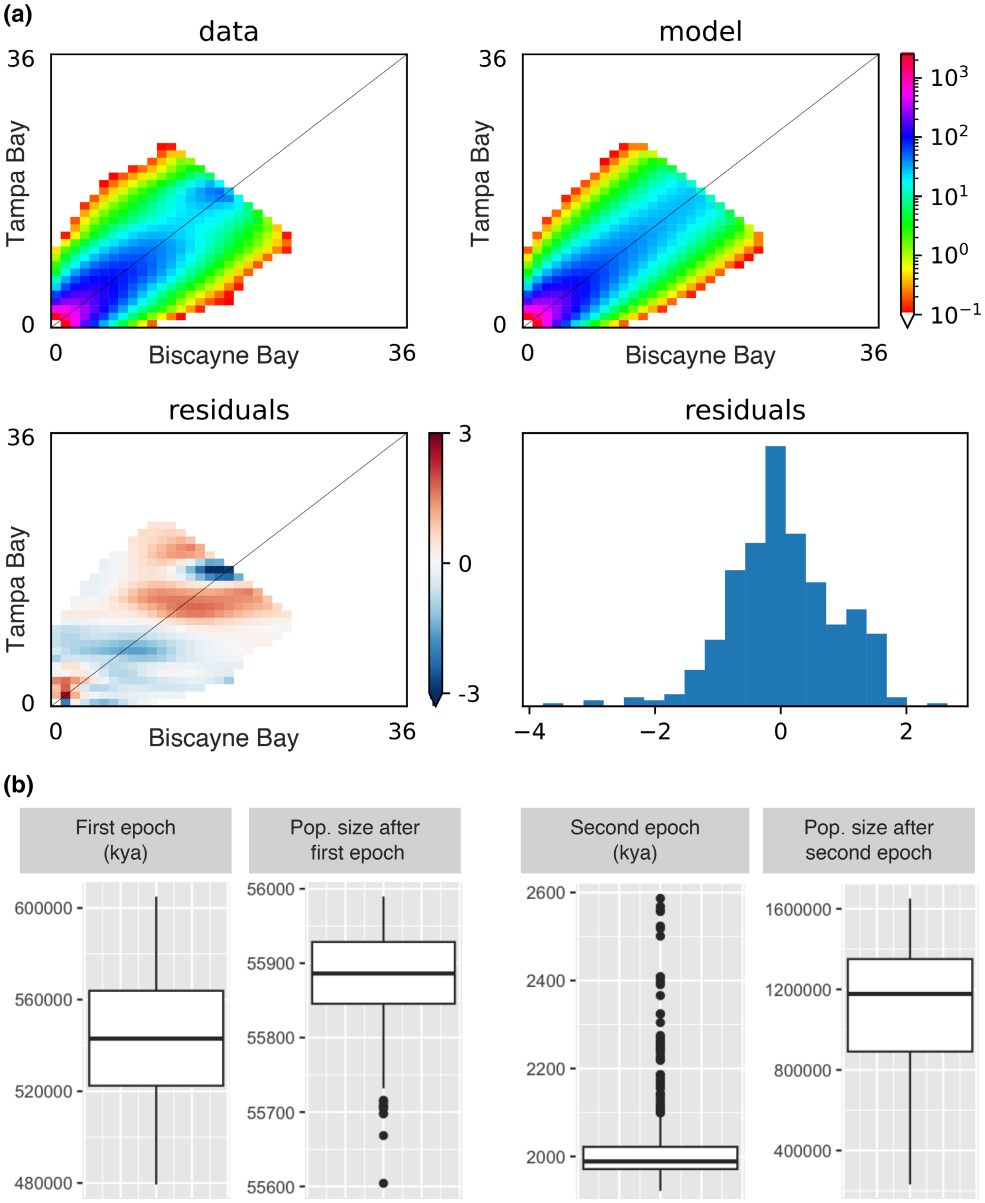
Best‐fit model among bootstrap replicates (sc2ns) and parameter uncertainties. (a, upper left) the 2D‐SFS averaged across bootstrap replicates. (a, upper right) The sc2ns model, which outperformed other simple models of population split and migration. Similar to Figure 2, the distribution of residuals (a, bottom) shows little difference between observed and predicted SFS. (b) Estimates of parameters and their uncertainties, derived from the sc2ns model on 100 bootstrapped SFS. Parameters of this model include the time since epochs of population growth and the effective population size at the end of each epoch.

To challenge our optimal model derived using GADMA, we conducted a second model selection by comparing seven simple demographic models on 10 bootstrapped SFS. The best‐fit model among the bootstrap replicates with the lowest median AIC score indicated two epochs of ancestral population growth and no population split (referred to as model “sc2ns” in Table 2 and Figure 3), which had 66% probability of being the best model by AIC weight. The small range of residuals between the observed and predicted SFS (Figure 3a) indicates a good fit of the sc2ns model to the data. However, we note that the sc2ns model appears to overestimate rare alleles from Tampa Bay and underestimate rare alleles from Biscayne Bay (bottom left corner of residual plot in Figure 3a), which possibly led to the conclusion that no population split occurred.

**TABLE 2:** Simple demographic models and their performance.

Model name	Model description	Median AIC	ΔAIC	Relative likelihood	AIC weight
sc2ns	No split, two epochs	1948.72	0	1	0.66
sc1ns	No split, one epoch	1950.75	2.03	0.36	0.24
sc3ns	No split, three epochs	1952.63	3.91	0.14	0.09
s2msm	One split, one epoch with symmetric migration	1968.08	19.36	6.25E‐05	4.15E‐05
sc2el	One split, two epochs with asymmetric migration	1998.72	50.00	1.39E‐11	9.24E‐12
s2m	One split, one epoch with asymmetric migration	2022.84	74.12	8.03E‐17	5.34E‐17
sc2elsm	One split, two epochs with symmetric migration	2261.93	313.21	9.72E‐69	6.46E‐69

*Note*: The winning model (sc2ns) is on top, and the remaining models are shown in order of increasing median AIC. Each model differs by whether the population splits, the number of epochs of population growth, whether split populations experience migration, and whether the migration is symmetric or asymmetric.

We ran the best‐fit sc2ns model on the 100 bootstrapped SFS to evaluate uncertainties around parameters of ancestral population size and theta (Figure S3) as well as epochs of ancestral population growth (Figure 3b). Based on these estimates, the first epoch likely occurred around 560 kya, after which the ancestral population grew from approximately 13,000 individuals to over 55,000 (Figure 3b). The second epoch of growth likely occurred around 2000 years ago, after which the population grew to approximately 1.2 million individuals (Figure 3b). Both of these epochs of population growth are in accordance with our best‐fit model from GADMA, although GADMA by design needed to infer a population split during the second growth epoch.

### 3.4 Genotype associations

We did not observe significant genetic differentiation associated with maturity among the whole sample set (*R* ^2^ = .0115; *p* = .29), or sex when evaluating only mature sharks (*n* = 67; *R* ^2^ = .0113; *p* = .597). Additionally, there were no genetic associations with morphology (i.e., precaudal length, fork length, and stretched total length) in a partial RDA conditional on sex and maturity (*R* ^2^ = 5.6e‐05; *p* = .503), and no significant variables were retained after forward selection. When evaluating female mature sharks (*n* = 36) and male mature sharks (*n* = 31) separately, no genetic associations with morphology were found (Females: *R* ^2^_adj_ = .0004, *p* = .39; Males: *R* ^2^_adj_ = .002, *p* = .083).

## 4 DISCUSSION

Our results suggest that bonnethead sharks from Tampa Bay and Biscayne Bay maintain high genetic connectivity. Although the two bays are significantly diverged in the ordination space (Figure 1b), more samples from Tampa Bay would improve our estimation of population divergence. Our first demographic inference using GADMA suggests that if the two populations diverged, it likely occurred as recently as 1500–4500 years ago. A previous study of bonnetheads from the Gulf and Atlantic coasts of Florida determined that populations diverged 15,000–34,000 years ago (Escatel‐Luna et al., 2015). We note that our study referenced the mutation rates of nuclear genes in the great hammerhead shark (*Sphyrna mokarran*), while the prior study referenced mutation rates of the mtDNA control region in the blacktip shark (*Carcharhinus limbatus*) and scalloped hammerhead (*Sphyrna lewini*). Consequently, these differences in mutation rates could affect our calculations by orders of magnitude. Nonetheless, these findings would be in accordance with previous literature documenting population divergence of bonnetheads in the Gulf of Mexico and Atlantic Ocean (Díaz‐Jaimes et al., 2021; Escatel‐Luna et al., 2015; Fields et al., 2016; Portnoy et al., 2015). However, although a population split between Tampa Bay and Biscayne Bay is supported by prior literature, our custom model selection procedure found that three demographic models with no split outperformed those with splits. Therefore, we are unable to definitively distinguish the two bays as distinct populations, and it is more likely that they represent a single continuous population through time.

While our multi‐model analysis involving both split and non‐split models did not support the split between our populations, it is notable that the model inferred by GADMA (log‐likelihood = −920.78) has a substantially better likelihood than the sc2ns model (log‐likelihood = −957.12) and all other models in the multi‐model run (see Tables 1 and 2), despite GADMA using the same input SFS. If the GADMA model was included in the AIC weight calculations shown in Table 2, it would outperform all other models. GADMA employs a sophisticated “genetic” algorithm to search the parameter space, which likely explains why it achieves a better solution than the original *moments* software that does not use such an algorithm. Additionally, we suspect the relatively higher performance of no‐split models in the custom multi‐model run may have resulted from ambiguous estimations of rare alleles. Specifically, the best‐fit sc2ns model seems to overestimate rare alleles in Tampa Bay while underestimating rare alleles in Biscayne Bay (Figure 3a). The proportion of rare alleles in each population influences the migration pattern inferred, as migrating individuals introduce rare alleles to the other population. More accurate estimates of these proportions would improve the rare alleles in our model and help determine if the populations are distinct and the direction of migration. Enhanced sampling, particularly in Tampa Bay, would aid in achieving these more accurate estimates.

On the other hand, both of our model selection procedures agreed that both bays experienced two epochs of population growth from ~10,000 individuals to over 1 million today. These findings suggest that bonnetheads in this region are increasing overall, which is concordant with another study that evaluated population growth using mtDNA (Escatel‐Luna et al., 2015). Management of bonnethead fisheries in the United States, Mexico, and Bahamas likely contributed to the population growth observed in these regions (IUCN, 2023), though unmanaged fishing pressure across the rest of their range exacerbates their decline (IUCN, 2023; Pérez‐Jiménez, 2014). The population growth observed here combined with the high connectivity potential (over 300 miles in this study) suggests that bonnetheads may not be as limited by dispersal as previously thought. With improved management across their range, recovery of genetic diversity in bonnethead populations is optimistic.

Although both models inferred similar rates of population growth, our GADMA model inferred a slower relative rate of population growth in Tampa Bay. It is possible, and perhaps likely, based on geography that the Biscayne Bay population also receives gene flow from more northern sites on the Atlantic coast to boost its effective population size (i.e., genetic diversity). Therefore, when considering shark conservation between the two sites, Tampa Bay may be a higher priority for evaluating population status. Marine life in Tampa Bay experiences threats such as nutrient discharge (Scolaro et al., 2023), red tide (Walsh et al., 2006), and bycatch (Hueter & Manire, 1994); however, many anthropogenic threats are also present in Biscayne Bay, including substantial urban development, pollution including eutrophication, and loss of seagrass and mangrove habitats (Cantillo et al., 2000). Although our findings suggest that the Biscayne Bay population is large and thriving, there is minimal data available on bonnethead sharks from Biscayne Bay and further research would be useful to quantify life history parameters in comparison with other populations.

In a previous genomic study of bonnetheads, Tampa Bay and Florida Bay did not exhibit population differentiation in neutral or outlier SNPs putatively under selection (Portnoy et al., 2015), suggesting that populations from Tampa Bay may migrate as far south as Florida Bay on the southwestern tip of Florida. Biscayne Bay in southeastern Florida is geographically close to Florida Bay (on the other side of the Florida Keys) but was not represented in prior genetic studies of bonnetheads. Our GADMA model predicts that populations from Tampa Bay may migrate beyond Florida Bay and across the Florida Keys to Biscayne Bay, a journey that is not geographically distant but presents a genetic barrier for many other marine species (Neigel, 2009). Therefore, these findings support the possibility that southern Florida may be a seasonal mixing zone (Ashe et al., 2015; Dimens et al., 2019; Kajiura & Tellman, 2016). Year‐round recapture studies could disentangle the migratory behavior of these populations, such as whether Biscayne Bay is a seasonal mating site between Gulf Coast and Atlantic populations.

While our results suggest that there is gene flow between Tampa Bay and Biscayne Bay, the signals within our genomic data (especially the unidirectional gene flow inferred by GADMA) may be driven by male‐mediated connectivity (Chapman et al., 2015). Studies evaluating maternally inherited mtDNA identified finer scale population structure than our findings here, likely driven by local adaptation of philopatric females (Díaz‐Jaimes et al., 2021; Escatel‐Luna et al., 2015; Fields et al., 2016; Portnoy et al., 2015). In a study combining mtDNA and genomic DNA, mtDNA demonstrated higher divergence than genomic DNA which was attributed to female philopatry and male‐biased dispersal (Portnoy et al., 2015). Thus, when considering male‐mediated movement as observed in other sharks (Ashe et al., 2015; Daly‐Engel et al., 2012; Dimens et al., 2019; Portnoy et al., 2015), our GADMA model's prediction of southward gene flow aligns with the idea that Tampa Bay males migrate southward to the Atlantic coast to mate with sharks from other bays (including Biscayne Bay females), but Tampa Bay females stay behind and mate locally. This hypothesis could be tested by examining genetic differences between male and female sharks but would be enhanced by a reference genome with annotated sex chromosomes.

Prior studies using genomic data also identified population divergence between the Gulf and Atlantic coasts, in regions more distant than those represented in this study (Díaz‐Jaimes et al., 2021; Portnoy et al., 2015). Those findings indicate that genetic differentiation may not be solely attributed to sex‐biased factors or female philopatry. Instead, this divergence could result from site fidelity in both sexes, possibly driven by seasonal feeding opportunities (Driggers et al., 2014). Additionally, these feeding behaviors may be learned and passed down to younger sharks through socially structured cognitive mapping (Driggers et al., 2014). Other factors that may drive genetic differentiation in sharks include local adaptation to environmental heterogeneity (Klein et al., 2024), food availability (Branham et al., 2022), or limited dispersal abilities, possibly due to habitat separation (Fields et al., 2016; Mendonça et al., 2011) changes in salinity (Ubeda et al., 2009). Since our findings show minimal genomic differentiation between Tampa Bay and Biscayne Bay, bonnetheads in these areas are likely not constrained by environmental adaptations or dispersal limitations. Future field studies could further investigate the roles of seasonal movements and socially structured cognitive mapping in driving these patterns.

In general, our findings suggest that bonnethead sharks from Tampa Bay and Biscayne Bay maintain high gene flow, although the specific direction of migration remains uncertain. Combined with previous observations of shark migrations, our best‐fit model from GADMA aligns well with the hypothesis that southern Florida may be a seasonal mixing zone. Though, expanding sampling efforts in Tampa Bay could refine our demographic models and clarify genomic differences between the bays. Additionally, tagging studies could verify the direction of shark migrations for mating. These findings enhance our understanding of migratory behavior of small coastal sharks and demonstrate that small‐bodied species may be less affected by geographic barriers to gene flow than others. Moreover, genetic connectivity between the Gulf of Mexico and Atlantic Ocean provides reason for optimism that under improved management bonnetheads may be capable of more rapid dispersal to overfished areas than previously thought. This shared genetic variation and naturally occurring gene flow will be useful for maintaining genetic diversity, preventing inbreeding, and aiding adaptation to future changes in either habitat.

## AUTHOR CONTRIBUTIONS

**Kristina L. Black:** Formal analysis (lead); funding acquisition (equal); investigation (lead); visualization (lead); writing – original draft (lead); writing – review and editing (lead). **Kathy Liu:** Data curation (equal); writing – review and editing (supporting). **Jasmin R. Graham:** Conceptualization (equal); data curation (equal); writing – review and editing (supporting). **Tonya R. Wiley:** Data curation (equal); writing – review and editing (supporting). **Jayne M. Gardiner:** Data curation (equal); funding acquisition (equal); writing – review and editing (supporting). **Catherine Macdonald:** Conceptualization (equal); data curation (equal); funding acquisition (equal); writing – review and editing (supporting). **Mikhail V. Matz:** Formal analysis (supporting); supervision (lead); writing – review and editing (supporting).

## FUNDING INFORMATION

Data collection in Biscayne Bay was supported by the Shark Research and Conservation Program at the University of Miami and Field School. Fieldwork in Tampa Bay was supported by a grant to J.M.G. from the Tampa Bay Environmental Restoration Fund. While this manuscript is based on data collected by J.M.G. while serving at the National Science Foundation, any opinions, findings, and conclusions or recommendations expressed in this material are those of the authors and do not necessarily reflect the views of the National Science Foundation. J.R.G. would also like to acknowledge the Maxwell/Hanrahan Foundation. Genetic analyses were supported by the NatureNet Science Fellowship from The Nature Conservancy to K.L.B.

## CONFLICT OF INTEREST STATEMENT

The authors declare no conflict of interest.

### OPEN RESEARCH BADGES

This article has earned an Open Data badge for making publicly available the digitally‐shareable data necessary to reproduce the reported results. The data is available at https://doi.org/10.5061/dryad.j3tx95xnq.

## Supporting information


Data S1:

## Data Availability

All sequences and metadata are deposited on the Sequence Read Archive under Bioproject: PRJNA1082259 (Accession nos.: SAMN40202348, SAMN40202454). All scripts and genotype data to reproduce the figures in this manuscript can be found on Dryad (https://doi.org/10.5061/dryad.j3tx95xnq).
