# Supplementary material for: Evidence for gene flow from the Gulf of Mexico to the Atlantic Ocean in bonnethead sharks (Sphyrna tiburo)
Source: Ecol Evol. 2024 Sep 22;14(9):e70334. doi: 10.1002/ece3.70334 (PMC11417010; doi:10.1002/ece3.70334)
Supplement: Supplementary file 1 — Data S1: [file ECE3-14-e70334-s001.docx]

**Supplemental Material**

**Supplementary section 1:** Genomic DNA isolation protocol

DNA was isolated using a combination of Qiagen Dneasy kit (ref) and a phenol-chloroform extraction protocol:

First, remove preservation agent (DMSO) from the tissue:

1. Cut fin clips into small tissue samples (~25mg) and place into 1.5ml tubes with 1000ml milliQ water. Centrifuge at 8000rpm for 30 seconds.
2. Discard the water and add 1000ml more milliQ water. Centrifuge for a second time at 8000rpm for 30 seconds.
3. Move the tissue to a clean dry tube and discard the first tube with water.

Follow the lysis steps of Qiagen protocol (using DNeasy Blood & Tissue kit) for purification of total DNA from animal tissues (spin-column protocol):

1. Add 180ul Buffer ATL and 20 ul proteinase K to sample tubes, and incubate tubes at 56C with frequent agitation until completely lysed.
2. Vortex the lysis, then centrifuge at 8000rpm for 30 seconds

The remaining steps follow a phenol-choloroform protocol:

1. Transfer the aqueous phase to a gel phase-lock tube and add 800ul chloroform/isoamyl alcohol (24:1) and vortex for a few seconds.
2. Place phase-lock tubes on ice for 1 minute. Then vortex again for 1-2 seconds.
3. Pipette the aqueous phase into a new 1.5ml microcentrifuge tube, then add 550ul ice cold isopropanol. Mix gently by inverting.
4. Incubate in a -20C freezer for 20 minutes
5. Centrifuge at max speed for 20 minutes at 4C.
6. Discard supernatant and add 1000ul 80% ETOH.
7. Centrifuge at max speed for 5 minutes at 4C.
8. Pour off supernatant and air dry the tubes upside down in a fume hood for 5 minutes.
9. Resuspend DNA in 30ul Buffer AE (from Qiagen kit).

The DNA extract is then cleaned using Zymo Genomic DNA Clean & Concentrator kit, following manufacturer’s instructions.

**
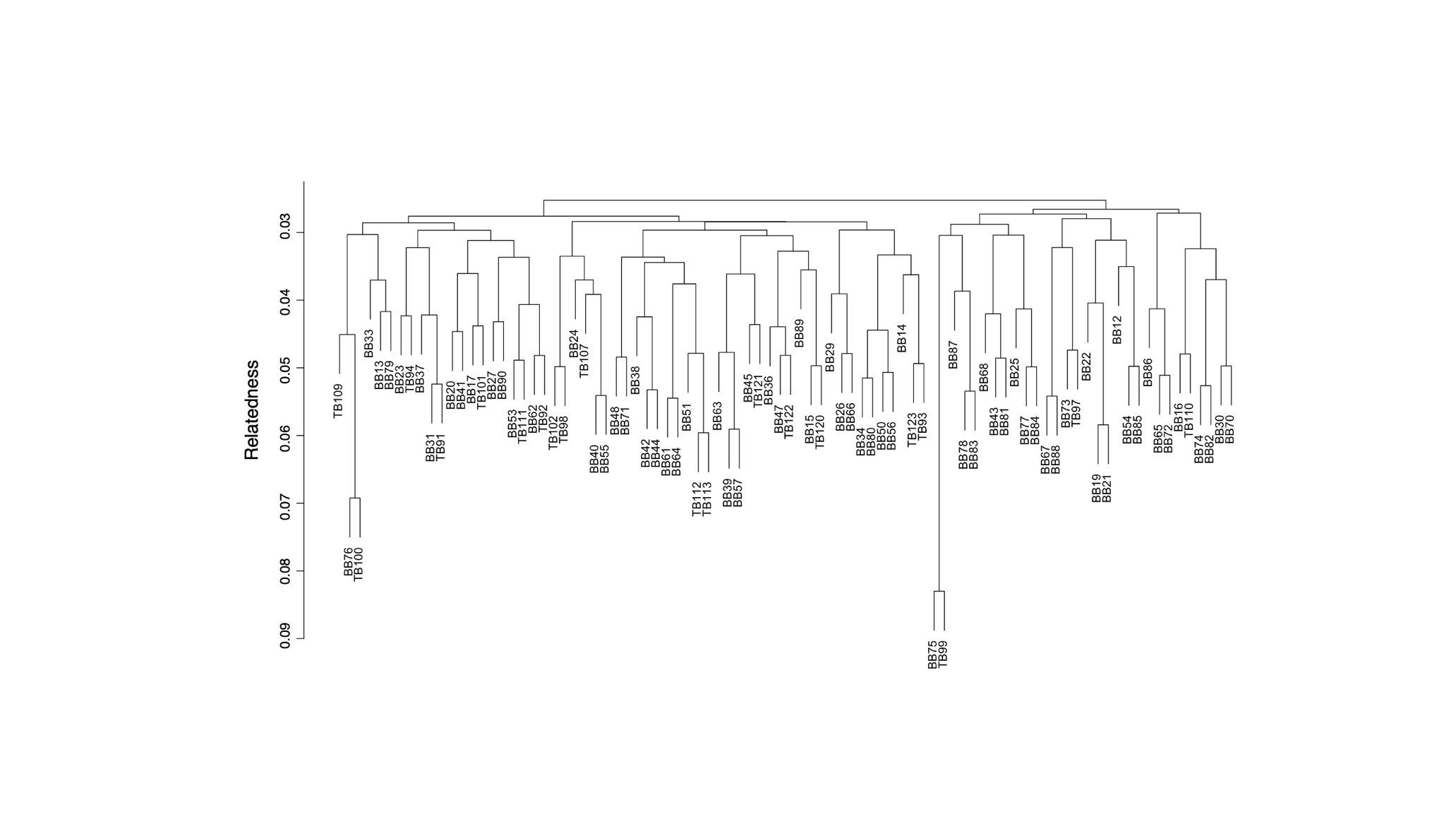
**

**Supplementary Fig. 1:** Relatedness among all bonnetheads sampled from Tampa Bay (TB) and Biscayne Bay (BB). All samples demonstrate low relatedness (<0.1) with each other. Low hanging branches near 0.5 would indicate 50 % allele sharing (such as full siblings), and samples near 1 would indicate identical twins.


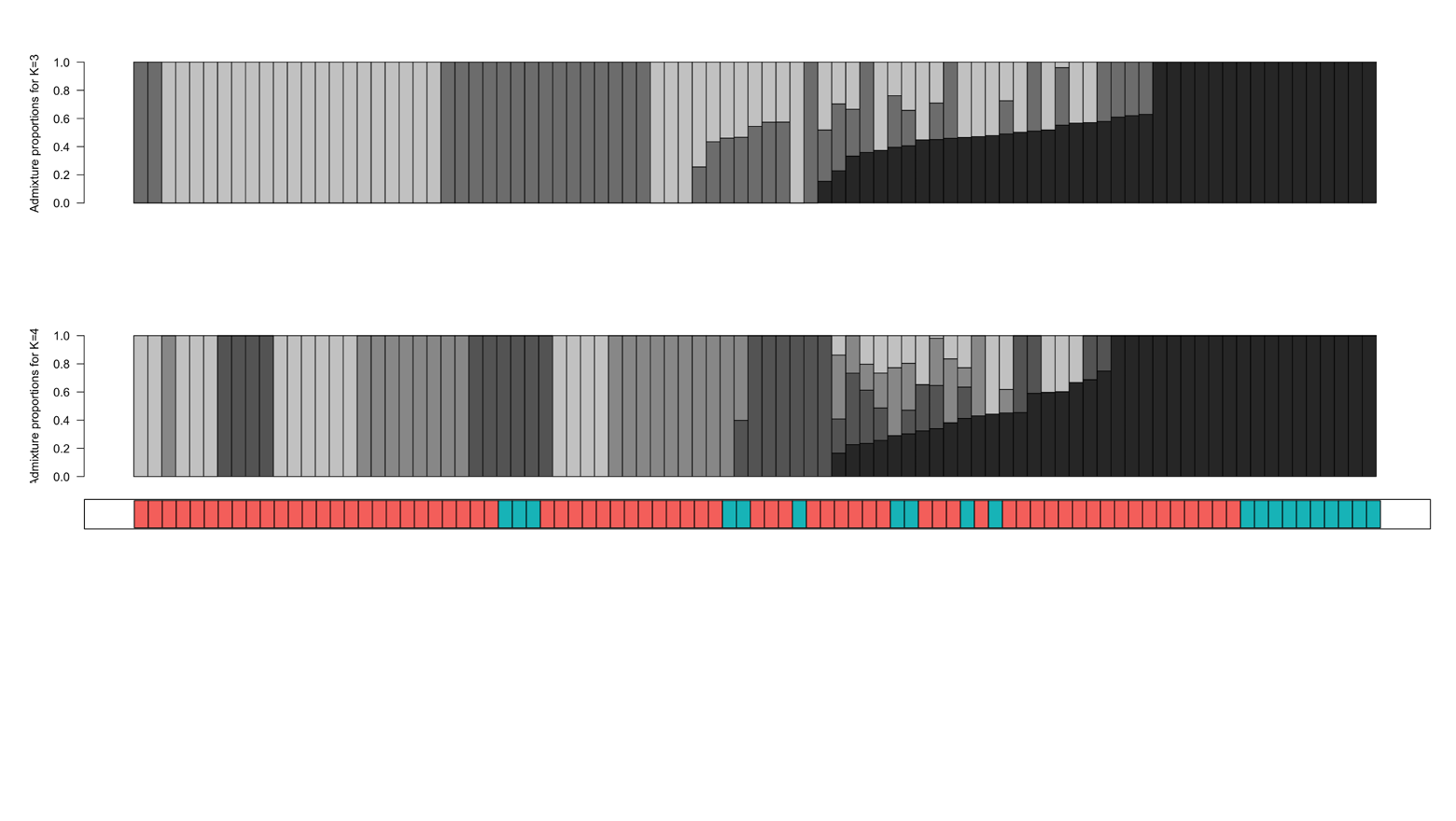


**Supplementary Fig. 2:** Admixture scenarios for k=3 (top) and k=4 (bottom). Samples from Tampa Bay are in red, and samples from Biscayne Bay are in blue.


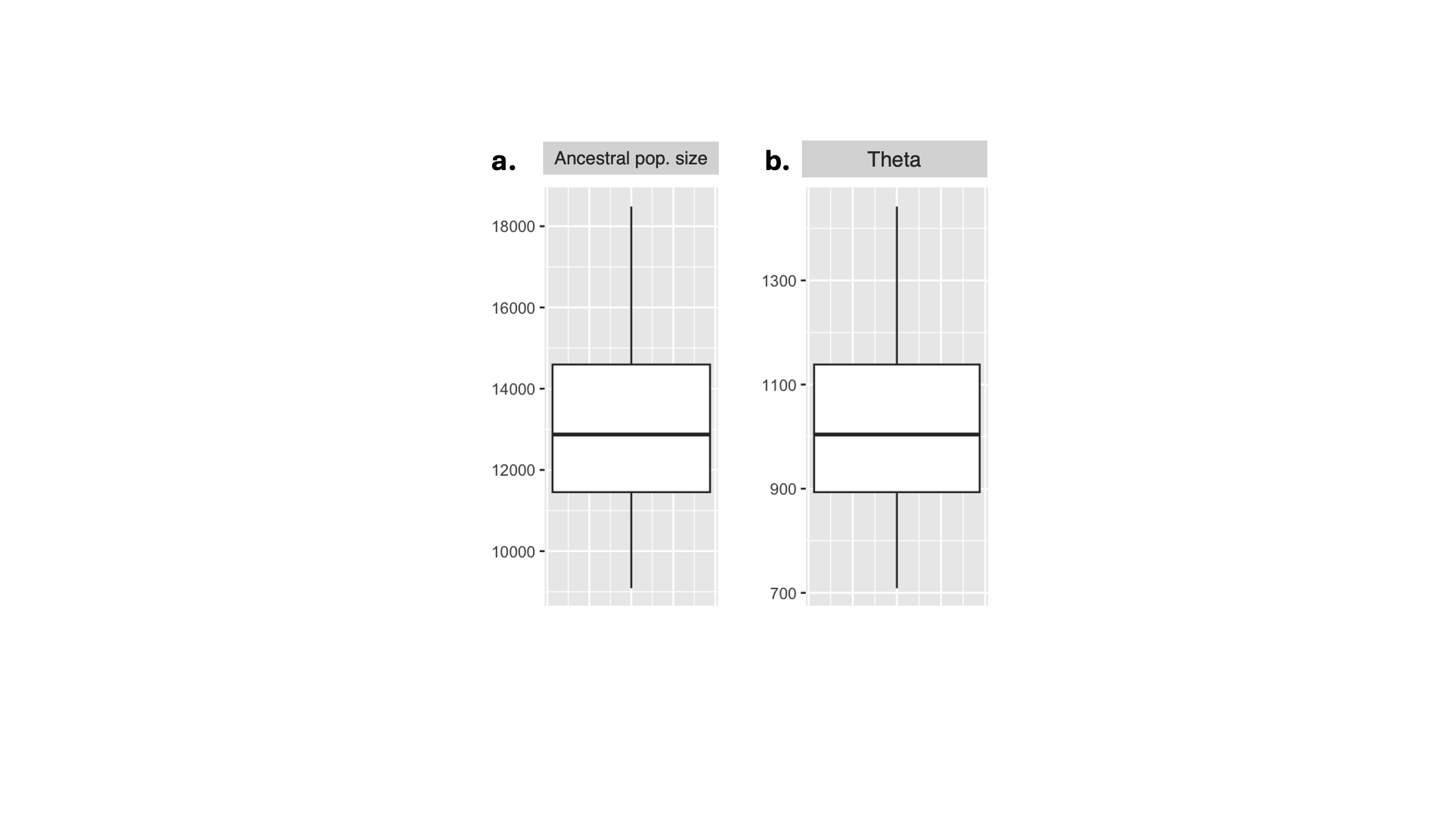


**Supplementary Figure 3:** Parameter uncertainties of ancestral population size (Ne) and theta (the population-size scaled mutation rate) as derived from a simple demographic model with two growth epochs and no population split (sc2ns) on 100 bootstrapped SFS.

**Supplementary Table 1:** Sequencing quality and population statistics for all 97 bonnethead samples before filtering. Samples starting with “BB” are from Biscayne Bay, and samples starting with “TB” are from Tampa Bay.

| Sample | Read  counts | Alignment rate | Heterozygosity | Inbreeding coefficient |
| --- | --- | --- | --- | --- |
| BB12 | 7227954 | 74.72 | 0.00115262 | 0.000762 |
| BB124 | 80543 | 43.71 | 0.00741425 | 0.001196 |
| BB13 | 4705218 | 74.85 | 0.00122303 | 0.000699 |
| BB14 | 3186503 | 75.28 | 0.00124051 | 0.001738 |
| BB15 | 7952267 | 75.38 | 0.00114723 | 0.002755 |
| BB16 | 2947034 | 74.52 | 0.00124041 | 0.003556 |
| BB17 | 7674959 | 75.29 | 0.00115436 | 0.001516 |
| BB18 | 1619668 | 62.77 | 0.00122424 | 0.18991 |
| BB19 | 3413459 | 75.22 | 0.00120685 | 0.003063 |
| BB20 | 2520194 | 75.08 | 0.00131091 | 0.00226 |
| BB21 | 2637101 | 74.54 | 0.0012419 | 0.004895 |
| BB22 | 5657129 | 74.81 | 0.00118197 | 0.001159 |
| BB23 | 4635880 | 74.74 | 0.00117687 | 0.000816 |
| BB24 | 2590962 | 73.13 | 0.00133282 | 0.002364 |
| BB25 | 2688183 | 75.27 | 0.00132952 | 0.00392 |
| BB26 | 3302300 | 75.04 | 0.00126945 | 0.00163 |
| BB27 | 2874155 | 75.21 | 0.00132516 | 0.00303 |
| BB28 | 586817 | 81.86 | 0.0154061 | 0.000429 |
| BB29 | 3201444 | 74.95 | 0.00120484 | 0.002423 |
| BB30 | 4064352 | 74.91 | 0.00120241 | 0.000961 |
| BB31 | 1136584 | 75.13 | 0.00124454 | 0.003134 |
| BB32 | 6497388 | 0.21 | 0.0164577 | 0.000032 |
| BB33 | 4838117 | 75.52 | 0.00118442 | 0.002105 |
| BB34 | 6215517 | 75.19 | 0.00112876 | 0.002197 |
| BB35 | 477528 | 70.65 | 0.00220222 | 0.015819 |
| BB36 | 3135592 | 74.82 | 0.00127801 | 0.000773 |
| BB37 | 4726188 | 74.88 | 0.00122624 | 0.000554 |
| BB38 | 6662845 | 75.43 | 0.00115698 | 0.001321 |
| BB39 | 3782707 | 75.44 | 0.00125306 | 0.000779 |
| BB40 | 3658637 | 75.03 | 0.00120319 | 0.001199 |
| BB41 | 6465290 | 75.34 | 0.0011695 | 0.000905 |
| BB42 | 3257828 | 74.7 | 0.00125706 | 0.001343 |
| BB43 | 5591972 | 75.45 | 0.00119846 | 0.001063 |
| BB44 | 3212265 | 74.5 | 0.00125908 | 0.00105 |
| BB45 | 6531780 | 75.22 | 0.0011564 | 0.001921 |
| BB47 | 5609902 | 75.33 | 0.00119761 | 0.000805 |
| BB48 | 6453334 | 75.04 | 0.00132173 | 0 |
| BB50 | 5502767 | 75.46 | 0.00119161 | 0.000626 |
| BB51 | 5104952 | 75.3 | 0.00121138 | 0.000708 |
| BB53 | 5477564 | 75.42 | 0.00116481 | 0.000671 |
| BB54 | 3523284 | 74.86 | 0.00126931 | 0.001411 |
| BB55 | 3614282 | 75.23 | 0.00118457 | 0.001478 |
| BB56 | 8916807 | 75.13 | 0.0011404 | 0.001048 |
| BB57 | 4391281 | 74.48 | 0.00120672 | 0.001024 |
| BB59 | 1877349 | 1.61 | 0.0125977 | 0.000129 |
| BB61 | 3184485 | 75.46 | 0.00123028 | 0.002316 |
| BB62 | 5733258 | 75.42 | 0.00116081 | 0.001043 |
| BB63 | 5245297 | 75.5 | 0.00116376 | 0.000655 |
| BB64 | 4956510 | 75.27 | 0.00115764 | 0.001074 |
| BB65 | 5715721 | 74.76 | 0.00119042 | 0.000629 |
| BB66 | 3687443 | 74.77 | 0.00123022 | 0.00234 |
| BB67 | 2516323 | 73.81 | 0.00123308 | 0.006832 |
| BB68 | 4872847 | 75.19 | 0.00119326 | 0.002077 |
| BB70 | 1351092 | 72.56 | 0.00117004 | 0.001878 |
| BB71 | 4505819 | 74.8 | 0.00121786 | 0.000404 |
| BB72 | 5018453 | 74.67 | 0.00116853 | 0.002044 |
| BB73 | 3880671 | 74.77 | 0.00129172 | 0.003062 |
| BB74 | 2898171 | 74.22 | 0.00122651 | 0.005489 |
| BB75 | 5517980 | 74.82 | 0.00118018 | 0.000813 |
| BB76 | 4247685 | 74.78 | 0.00118409 | 0.001981 |
| BB77 | 5821997 | 74.82 | 0.00117204 | 0.001656 |
| BB78 | 5026659 | 75.32 | 0.00123726 | 0.00066 |
| BB79 | 6654406 | 75.18 | 0.00117805 | 0.001648 |
| BB80 | 2740571 | 74.91 | 0.00147879 | 0.001802 |
| BB81 | 2383096 | 74.94 | 0.0013394 | 0.003682 |
| BB82 | 1736298 | 73.64 | 0.00120862 | 0.013857 |
| BB83 | 4624638 | 75.12 | 0.00122147 | 0.001986 |
| BB84 | 3149876 | 75.15 | 0.00118744 | 0.003888 |
| BB85 | 4875977 | 75.48 | 0.00118076 | 0.001941 |
| BB86 | 1962196 | 74.25 | 0.00128702 | 0.008651 |
| BB87 | 3735927 | 74.54 | 0.00124607 | 0.001873 |
| BB88 | 4608407 | 74.94 | 0.00117033 | 0.001686 |
| BB89 | 10768328 | 75.1 | 0.00110958 | 0.001616 |
| BB90 | 7706384 | 75.29 | 0.0011632 | 0.000902 |
| TB91 | 2965119 | 74.54 | 0.00128839 | 0.001255 |
| TB92 | 6269896 | 75.22 | 0.00115664 | 0.000772 |
| TB93 | 5206574 | 75.53 | 0.00120411 | 0.000541 |
| TB94 | 3231335 | 74.65 | 0.00120849 | 0.001987 |
| TB95 | 490175 | 62.81 | 0.00157252 | 0.020525 |
| TB96 | 169504 | 59.54 | 0.00285411 | 0.004511 |
| TB97 | 3778051 | 75.66 | 0.00120245 | 0.005371 |
| TB98 | 2619833 | 75.02 | 0.00127817 | 0.004513 |
| TB99 | 13986853 | 74.93 | 0.00111658 | 0.002095 |
| TB100 | 10792878 | 75 | 0.00113479 | 0.000438 |
| TB101 | 6086436 | 75.25 | 0.001155 | 0.001012 |
| TB102 | 6176998 | 75.3 | 0.00115087 | 0.000763 |
| TB107 | 2366804 | 74.56 | 0.00124411 | 0.004829 |
| TB109 | 4860019 | 74.91 | 0.00120283 | 0.000683 |
| TB110 | 8212585 | 75.33 | 0.00112211 | 0.001691 |
| TB111 | 8858909 | 75.41 | 0.00113681 | 0.001403 |
| TB112 | 4377159 | 75.72 | 0.00118631 | 0.001105 |
| TB113 | 4375942 | 75.84 | 0.00119774 | 0.000644 |
| TB120 | 3672127 | 76.02 | 0.00122171 | 0.001305 |
| TB121 | 6707738 | 75.92 | 0.00114993 | 0.001302 |
| TB122 | 3385992 | 75.73 | 0.00124178 | 0.00105 |
| TB123 | 8278152 | 75.73 | 0.00114165 | 0.000487 |

**Supplementary Table 2:** Ancestral population growth as estimated by the top five best-fit models by GADMA.

| **Log-likelihood** | **Years since epoch of ancestral population growth** | **Estimated ancestral Ne after epoch of ancestral population growth** |
| --- | --- | --- |
| -920.78 | 410,528 | 51,586 |
| -920.81 | 408,330 | 51,611 |
| -920.86 | 380,818 | 51,611 |
| -920.86 | 382,035 | 51,636 |
| -920.93 | 382,035 | 51,736 |
